# Weighted Attribute-Based Proxy Re-Encryption Scheme with Distributed Multi-Authority Attributes

**DOI:** 10.3390/s24154939

**Published:** 2024-07-30

**Authors:** Wenlong Yi, Chuang Wang, Sergey Kuzmin, Igor Gerasimov, Xiangping Cheng

**Affiliations:** 1 School of Software, Jiangxi Agricultural University, Nanchang 330045, China; yiwenlong@jxau.edu.cn (W.Y.); wangchuang@stu.jxau.edu.cn (C.W.); 2 Faculty of Computer Science and Technology, Saint Petersburg Electrotechnical University “LETI”, Saint Petersburg 197022, Russia; ksa84@yandex.ru (S.K.); ivgerasimov-45@yandex.ru (I.G.); 3 Institute of Applied Physics, Jiangxi Academy of Sciences, Nanchang 330096, China

**Keywords:** data sharing, access control, blockchain, data security, attribute authority

## Abstract

Existing attribute-based proxy re-encryption schemes suffer from issues like complex access policies, large ciphertext storage space consumption, and an excessive authority of the authorization center, leading to weak security and controllability of data sharing in cloud storage. This study proposes a Weighted Attribute Authority Multi-Authority Proxy Re-Encryption (WAMA-PRE) scheme that introduces attribute weights to elevate the expression of access policies from binary to multi-valued, simplifying policies and reducing ciphertext storage space. Simultaneously, the multiple attribute authorities and the authorization center construct a joint key, reducing reliance on a single authorization center. The proposed distributed attribute authority network enhances the anti-attack capability of cloud storage. Experimental results show that introducing attribute weights can reduce ciphertext storage space by 50%, proxy re-encryption saves 63% time compared to repeated encryption, and the joint key construction time is only 1% of the benchmark scheme. Security analysis proves that WAMA-PRE achieves CPA security under the decisional q-parallel BDHE assumption in the random oracle model. This study provides an effective solution for secure data sharing in cloud storage.

## 1. Introduction

**Figure 1:**
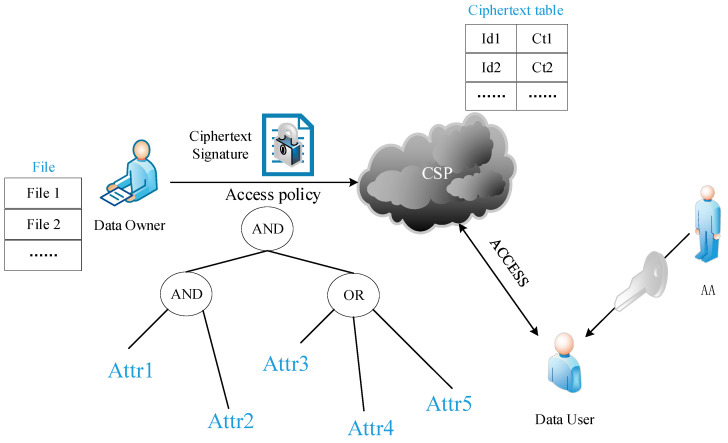
Access control schema for cloud storage data.

With technological advancements, internet services are becoming increasingly personalized, open, intelligent, and transparent. Under the driving forces of new technologies like cloud computing, big data, the Internet of Things, and artificial intelligence, user information is exhibiting explosive growth [1]. In an open, transparent, and interconnected environment, information security, integrity, confidentiality, availability, and ownership are critical [2]. However, traditional computer information storage and sharing methods impose high hardware and software requirements and cause many inconveniences due to low storage efficiency and cumbersome management. To meet the demand for large-capacity storage and sharing, more and more users are opting for cloud storage service platforms based on data centers [3]. Nevertheless, cloud storage may face security risks such as information leakage, and it is challenging to ensure the integrity, accuracy, and confidentiality of information [4,5,6]. The application of attribute-based encryption technology effectively resolves the limitation of traditional public key encryption in data sharing, where access is either fully authorized or completely prohibited, enabling the development of finer-grained access control [7]. Fine-grained access control ensures secure data sharing in multi-user and big-data scenarios. By specifying access policies, it allows only users with specific attributes to gain data access privileges. As illustrated in Figure 1, in a cloud storage system incorporating attribute-based encryption (ABE), the data owner encrypts the data file (File) using an encryption algorithm based on a specific access policy, generating ciphertext (Ct1, Ct2) with the access policy embedded within it. Subsequently, the data owner signs the ciphertext and transmits it in encrypted form for storage with the cloud service provider (CSP). The CSP maintains a table (ciphertext table) containing ciphertext identifiers (Id1, Id2) to facilitate user searches. Users retrieve the corresponding ciphertext from the CSP and attempt decryption using private keys generated by the Attribute Authority (AA). Decryption is successful if the user’s attributes (Attr) satisfy the access policy embedded in the ciphertext; otherwise, decryption fails.

Currently, most cloud storage sharing systems adopt a centralized management model, where the cloud service provider (CSP) centrally manages all data. If the CSP encounters hardware or software malfunctions or is attacked, it could lead to information loss, leakage, or service interruption. In contrast, blockchain, as a decentralized, immutable, and unforgeable distributed ledger technology, provides a new option for information security [8]. Blockchain ensures information integrity, non-repudiation, privacy, and tamper-resistance through decentralized storage, P2P transmission, smart contracts, consensus mechanisms, and encryption techniques. It packages information into blocks, chains them chronologically using a specific data structure, validates and stores the information through a consensus mechanism, and uses encryption algorithms to ensure secure information transmission and interaction. Smart contracts can automatically control system execution, reducing intermediaries and increasing operational transparency. Blockchain technology meets the modern demands for sharing, openness, fair competition, authenticity, integrity, security, and reliability [9]. Based on the differences in participants and consensus mechanisms, blockchain can be categorized into public, consortium, and private chains [10]. Public chains are open blockchains, where anyone can participate in network transactions and the consensus process. Public chains typically employ consensus mechanisms such as Proof of Work (PoW), which require substantial mining efforts to validate transactions and generate new blocks. Consortium chains are blockchains jointly managed by multiple pre-selected institutions or organizations. Only authorized nodes can participate in the consensus process. Consortium chains generally utilize the Raft algorithm or other lightweight consensus algorithms, eliminating the need for computationally intensive mining operations. Consortium chains balance performance and security, making them suitable for cross-organizational data sharing and collaboration. Private chains are blockchains entirely controlled by a single organization or institution.

Many scholars have achieved data access control in various scenarios by combining attribute-based encryption and blockchain technologies [11,12,13,14,15,16]. However, existing schemes have the following issues: implementing traditional ciphertext-policy attribute-based encryption, which does not allow for the modification of access policies, necessitates data owners to re-encrypt and re-store information on the blockchain, resulting in the accumulation of multiple encryptions and redundant data on the chain; relying on a centralized server for access control and authorization, which is prone to single-point failure; the limited expressiveness of access policies represent only the “satisfaction” or “non-satisfaction” of single attributes, resulting in complex access policies, large ciphertext sizes, and high encryption time costs as the number of attributes in the access policy increases. This study proposes a Weighted Attribute Authority Multi-Authority Proxy Re-Encryption (WAMA-PRE) with the following main contributions:

1. Incorporating blockchain and attribute-based proxy re-encryption achieves fine-grained data access control and storage segregation, transferring access control from the centralized CSP to a decentralized blockchain for enhanced data security.
2. It improves traditional algorithms by proposing a joint key generation algorithm involving multiple authorities and authorization centers, mitigating a single authorization center’s single-point failure.
3. It proposes weighted attribute representation for access policies, addressing the single attribute “satisfaction/non-satisfaction” limitation, simplifying policies, reducing ciphertext space, and improving encryption speed.
4. Experimental validation of the WAMA-PRE scheme’s storage and time efficiency performance. The scheme’s robust security against chosen-plaintext attacks is also verified under the random oracle model.

This paper comprises six sections. The Section 1, the Introduction, presents the research background, articulates the research issue, and outlines the study’s contributions. The Section 2, Related Work, reviews the current state of ABE research and its optimization schemes, identifying areas requiring further investigation. The Section 3, the Method, describes the proposed model design, algorithm design, and operational procedures. The Section 4, the Results, describes the experimental environment and presents the experimental outcomes of the proposed model. The Section 5, the Discussion, offers a quantitative analysis of the proposed model’s performance and provides a security proof. The Section 6, Conclusions, summarizes the research and presents future research prospects.

## 2. Related Work

Sahai et al. [17] adopted a fuzzy identity-based encryption approach and first proposed the concept of ABE, which has since seen substantial development and produced many critical solutions. Current attribute-based encryption schemes are mainly divided into two categories: one is Key-Policy Attribute-Based Encryption (KP-ABE) [18], and the other is ciphertext-policy attribute-based encryption (CP-ABE) [19]. Compared to KP-ABE, CP-ABE allows data owners to define flexible access policies, better meeting the data sharing needs in cloud storage, thus promoting the proposal of various CP-ABE schemes.

Wang et al. [20] proposed a file hierarchy attribute-based encryption scheme that utilizes an integrated access structure to encrypt hierarchical files, achieving secure access control for hierarchical shared data. Li et al. [21] proposed a searchable CP-ABE scheme with attribute revocation, preventing receivers from extracting sensitive information from the ciphertext by partially hiding the access structure while realizing attribute revocation and key updates. Feng et al. [22] introduced searchable encryption into attribute-based encryption, proposing a scheme supporting direct user revocation, where a central authority controls access to avoid the security risks of submitting private keys and access structures to the cloud server. Ge et al. [23] introduced data integrity protection into revocable attribute-based encryption and verified its confidentiality and integrity. This line of work shifts security risks to the central authority, raising the issue of how to verify the security of the central authority. To address this, Yang et al. [24] proposed a revocable CP-ABE scheme that delegates ciphertext updates and re-encryption to a semi-trusted third party, such as a cloud service provider, providing backward and forward secrecy. Zhang et al. [25] proposed a CP-ABE scheme supporting partial access structure hiding and key revocation, constructing the access structure using linear secret sharing and supporting “AND” and “OR” gate operations for access policies, making encryption and decryption control more flexible.

These attribute-based encryption schemes address the fine-grained access control and transmission confidentiality of cloud storage data to some extent. However, they still need to improve on their low efficiency, inflexible data sharing and delegation operations, inability to update permissions in real-time, and data availability issues. To solve these problems, researchers introduced proxy re-encryption techniques [26], which allow data owners to delegate data access rights to a proxy, enabling more flexible permission management without sharing decryption keys. It makes secure data sharing possible in distributed environments and improves the availability of cloud storage data, protecting data even if the cloud service provider is attacked.

Since the first proxy re-encryption scheme was proposed, proxy re-encryption schemes have made substantial progress over the past decade: schemes based on user identities rather than public keys [27] simplify the public key certificate verification of identity-based encryption but require explicit specification of receivers. Conditional ciphertext transformation based on identity-based encryption realizes partial decryption permission delegation but still in a one-to-one form [28]. Liang et al. [29] extended the conditions and identity descriptions based on proxy re-encryption and attribute encryption, proposing an attribute-based proxy re-encryption scheme and proving its chosen-plaintext security and master key security. Luo et al. [30] designed an attribute-based proxy re-encryption scheme supporting multi-valued negative attributes and wildcards, achieving master key security and access structure control. Concurrently, Mizuno and Doi [31] first proposed an attribute-to-identity mixed scheme. Attribute-based proxy re-encryption integrates the one-to-many access control of attribute-based encryption and the data delegation advantage of proxy re-encryption. However, it relies on a single authorization center, affecting security and efficiency. Introducing multiple attribute authorities [32,33,34] mitigates the security risks associated with centralized authorization. Therefore, Liu et al. [35] designed a Multi-Authority CP-ABPRE (MA-CP-ABPRE) scheme, replacing the single authorization center with multiple authority centers.

However, in terms of attribute matching, the access policies in existing schemes mainly use binary “satisfaction” and “non-satisfaction” representations of attributes, which are unable to accurately express the degree of attribute matching. Considering the different importance of attributes, researchers have proposed Ciphertext-Policy Weighted Attribute-Based Encryption (CP-WABE) schemes [36,37]. Fan et al. [38] proposed a scheme supporting multi-state attribute expressions, not only binary states, making attribute expressions more flexible and supporting dynamic joining and updating. Wang et al. [39] introduced the concept of weighted attributes, allowing an extension from binary to any state expression and reducing the complexity of access policies. Additionally, schemes supporting range attributes [40,41,42], such as time, location, and numerical ranges, provide more representative policy expressions.

The abovementioned schemes attempt to improve the accuracy of access policy attribute expressions through attribute refinement, state mapping, weight assignment, and other techniques, enhancing attribute expression capabilities to some extent. However, issues such as low efficiency or limited expression capability persist, with further improvement especially needed in the attribute representation within access policies.

## 3. Method

### 3.1. Model Design

**Figure 2:**
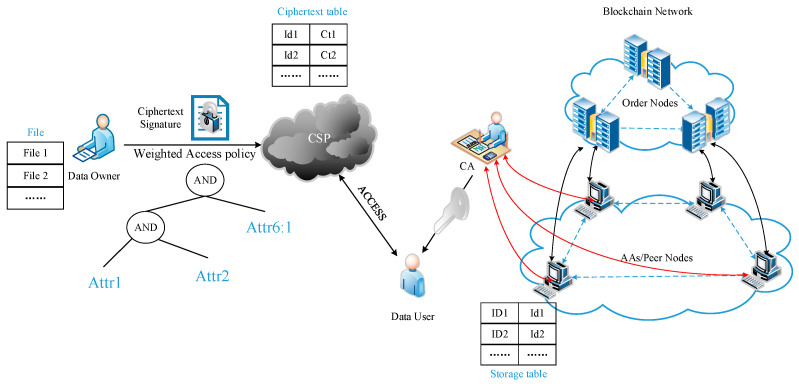
WAMA-PRE architecture.

This study proposes a WAMA-PRE scheme for distributed multi-attribute authorities, as illustrated in Figure 2. The system comprises the following key components:

Central Authority (CA): The CA is responsible for system initialization, receiving user key components generated by attribute authorities, and generating user keys. It registers each user and maintains a list containing user details to verify user authenticity.

Attribute Authority (AA): Each AA is responsible for generating private and public key pairs for the set of attributes within its domain. An AA can manage multiple attributes, but each attribute is managed by only one AA. AAs also generate user key components related to users’ attributes.

Data Owner (DO): The DO has absolute control over their shared data and can customize data access permissions, enabling fine-grained access control. Before uploading data to the cloud storage system, the DO encrypts the data using a defined weighted access policy.

Data Requester (DR): DRs consist of authorized and unauthorized users. Authorized users can decrypt ciphertexts using their attribute private keys. Unauthorized users gain access permissions by sending data-sharing requests to authorized users. When an unauthorized user wants to access encrypted data, an authorized user, acting as a data-sharing authorizer, is responsible for reviewing the data-sharing request. They generate a re-encryption key and send it to the orderer node cluster if approved.

Cloud Service Provider: the cloud service provider is responsible for storing ciphertexts uploaded by data owners and maintaining a ciphertext table.

Orderer Node Cluster: The orderer node cluster is crucial to ensuring transaction order consistency in the blockchain system. In this scheme, orderer nodes act as third-party proxies executing proxy re-encryption operations, modifying existing ciphertexts’ access policies, and recording re-encryption operations on the blockchain.

Blockchain Network: The blockchain network stores transactions and metadata of shared data. The metadata includes hash values of encrypted data in cloud storage. When data requesters download encrypted data from cloud storage, they can verify the hash values to ensure the integrity of the encrypted data.

Furthermore, the cryptographic symbols involved in the WAMA-PRE model scheme are presented in Table 1.

### 3.2. Algorithm Design

In the WAMA-PRE architecture, the proposed algorithms mainly include *GlobalSetup*, *AASetup*, *KeyGen*, *Encrypt*, *ReKeyGen*, *ReEncrypt*, *Decrypt*, and *ReDecrypt*, consisting of eight phases as follows:

1. *GlobalSetup*(1*^k^*) → *MPK,MSK*. Taking the security parameter 1*^k^* as input, it outputs the system public key *MPK* and the system master key *MSK*.
2. *AASetup*(*MPK*, *U_i_*) → *PK_i,j_*,*SK_i,j_*. Taking the system public key *MPK* and the attribute set *U_i_* managed by the attribute authority *AA_i_* as input, it generates the attribute public key *PK_i,j_* and the attribute private key *SK_i,j_* for each attribute *attr_j_* in *U_i_*.
3. *KeyGen*(*MSK*, *USK_i_*, *S*) → *USK*. Taking the system master key *MSK*, user key component *USK_i_*, and attribute set *S* as input, it outputs the user key *USK* corresponding to the attribute set *S*.
4. *Encrypt*(*MPK*, (*M*, *ρ*), *m*) → *CT*. Taking the system public key *MPK*, weighted access structure (*M*, *ρ*), and plaintext m as input, it outputs the ciphertext *CT*.
5. *ReKeyGen*(*MPK*, *USK*, (*M*′,*ρ*′)) → *RK_S→_*_(*M*_*_′,ρ__′_*_)_. Taking the system public key *MPK*, user key *USK*, and weighted access structure (*M*^′^,*ρ*^′^) as input, it outputs the re-encryption key *RK_S→_*_(*M*_*_′,ρ__′_*_)_.
6. *ReEncrypt*(*MPK, RK_S→_*_(*M*_*_′,ρ__′_*_)_, *CT*) →*CT’*. Taking the system public key *MPK*, re-encryption key *RK_S→_*_(*M*_*_′,ρ__′_*_)_, and ciphertext *CT* as input, if the attribute set *S* corresponding to the user key *USK* satisfies the minimum weight of the access structure, i.e., *S* | = (*M*, *ρ*), it outputs the re-encrypted ciphertext *CT*′; otherwise, it outputs ⊥, indicating decryption failure.
7. *Decrypt*(*MPK*, *USK*, *CT*) → *m*. Taking the system public key *MPK*, user key *USK*, and ciphertext *CT* as input, if *S* | = (*M*, *ρ*), it outputs *m*; otherwise, it outputs ⊥.
8. *Decrypt_R_*(*MPK*,*USK*,*CT′*) → *m*. Taking the system public key *MPK*, user key *USK*, and re-encrypted ciphertext *CT*′ as input, if *S*′ | = (*M*′,*ρ*′), it outputs the plaintext information m; otherwise, it outputs ⊥.

### 3.3. WAMA-PRE Execution Policy

The WAMA-PRE process mainly includes four stages: system initialization, data encryption and ciphertext on-chaining, data ciphertext retrieval and decryption, and ciphertext re-encryption.

1. System Initialization. In the blockchain system, the CA first executes the *GlobalSetup* function, taking the security parameter 1*^k^* as input, and selects two cyclic groups, *G* and *G_T_*, of prime order *p*, where $g_{1}$ and $g_{2}$ are generators of the group *G*. It randomly chooses $a_{0},a_{1},a_{2}\in Z_{p}^{*}$, and $e:G\times G\to G_{T}$ is a bilinear map. The hash functions are $H_{1}:{\left\{0,\;1\right\}}^{*}\to G$ and $H_{2}:G_{T}\to Z_{p}^{*}$, resistant to collusion. Equations (1) and (2) show that it outputs the system master key *MSK* and the system public key *MPK*.
  (1) $$MSK=\left\{a_{1},a_{2}\right\}$$
  (2) $$MPK=\left\{p,G,G_{T},g_{1},g,g^{a_{0}},e,H_{1},H_{2},e{\left(g,g\right)}^{a_{1}},g^{a_{2}}\right\}$$
  In the blockchain network, the attribute universe is $U=\{attr_{1},\;attr_{2},\;\ldots ,\;attr_{j}\}$, and the weight set is $W=\;\{w_{1},\;w_{2},\;\ldots ,\;w_{n}\}$, where *attr_j_* represents the *j*-th attribute, and $w_{n}$ represents the *n*-th weight. Therefore, this blockchain network contains *j* × *n* weighted attributes, obtaining the weighted attribute set $A=\{attr_{1}:w_{1},\ldots ,\;attr_{1}:w_{n},\ldots ,\;attr_{j}:w_{1},\ldots ,\;attr_{j}:w_{n}\}$. Each *AA* manages a subset of attributes. Let the current attribute authentication center be *AA_i_*. *AA_i_* executes the *AASetup* function, taking the system public key *MPK* and the attribute set *U_i_*, managed by the attribute authority *AA_i_*, as input. As shown in Equation (3), the attribute authority *AA_i_* randomly selects $h_{i,j}\in Z_{p}^{*}$ as the private key *SK_i,j_* for each attribute $attr_{j}$, and then it generates the attribute public key $PK_{i,j}$ as shown in Equation (4).
  (3) $$SK_{i,j}=\{h_{i,j}\}$$
  (4) $$PK_{i,j}=\{g^{a_{0}h_{i,j}},g^{h_{i,j}},g^{a_{2}h_{i,j}}\}$$
  when new users join the blockchain system, they first register their identity information with the CA, including their attribute set *S* and personal information. The CA assigns the user a global user identifier *GID* and then sends a key construction request to the corresponding *AA*. After receiving the request, the *AA* generates the user key component *USK_i_* based on the user’s attribute information, as shown in Equation (5).
  (5) $$USK_{i}=\{K_{i}=g^{a_{0}h_{i}},L_{i}=g^{h_{i,}},T_{i}=g^{a_{2}h_{i}}\}$$
  where $h_{i}=h_{i,1}+h_{i,2}+h_{i,3}+\ldots +h_{i,j}$, j∈S. After receiving the user key component, the central authority runs the *KeyGen* algorithm, as shown in Equation (6), to generate the user key *USK* for the user.
  (6) $$USK=\{S,K=g^{a_{0}h}\cdot g^{a_{1}},L=g^{h},T_{x}=g^{a_{2}h}\}$$
  where $h=h_{1}+h_{2}+h_{3}+\ldots +h_{k}$, and *k* is the number of involved attribute authorities; the attribute key *USK* is then sent to the user through a secure channel for storage.
2. Data Encryption and Ciphertext On-Chaining. For the data file *File* of the DO in the blockchain network, a globally unique file number *UFID* is generated. A random number $\varepsilon \in G_{T}$ is chosen, where *G_T_* is a cyclic group of prime order *p*, and the symmetric key $key=H_{2}(\varepsilon )$ is generated. The symmetric encryption algorithm $E_{key}$ is run, taking the symmetric key *key* and the data file *File* as input to generate the data ciphertext *CF*.

**Figure 3:**
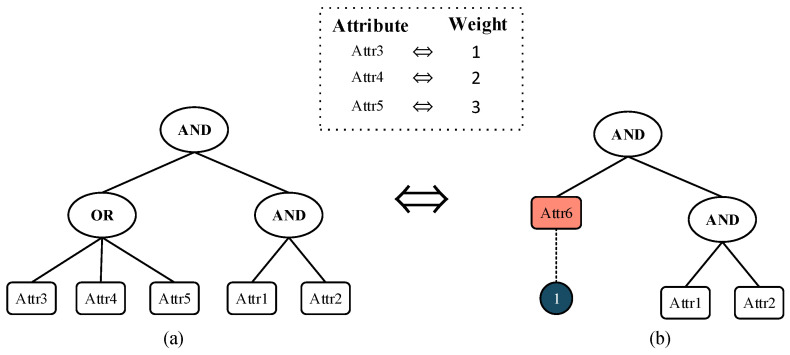
Access policies. (**a**) Standard access policy *T*; (**b**) weighted access policy *WT*.

The DO selects appropriate attributes to formulate an access policy *T*, a Boolean expression containing “*AND*”, “*OR*”, and attributes. For example, T{(“Attr3” OR “Attr4” OR “Attr5”) AND (“Attr1” AND “Attr2”)}. As illustrated in Figure 3, the access policy *T* is converted into a weighted access policy *WT*. Suppose the attribute weights for *Attr3*, *Attr4*, and *Attr5* are set to 1, 2, and 3, respectively. These three attributes can be represented by a single attribute, “*Attr6*”, with different weights, denoted as “*Attr6*:1”, “*Attr6*:2”, and “*Attr6*:3”. It can be any state attribute, such as “*Attr6*:*Attr3*, *Attr4*, *Attr5*”. Consequently, the access policy T{(“Attr3” OR “Attr4” OR “Attr5”) AND (“Attr1” AND “Attr2”)} can be transformed into WT{“Attr6:1” AND (“Attr1” AND “Attr2”)}.

In the weighted access policy, “*Attr6*:1” represents the minimum threshold of 1 that needs to be met, implicitly including “*Attr6*:1”, “*Attr6*:2”, and “*Attr6*:3”. Compared to the access policy *T*, the weighted access policy *WT* reduces the number of attributes by two. Therefore, *WT*’s representation is more flexible and concise. During ciphertext computation, this approach will also decrease the number of attributes, thereby reducing storage space utilization.

The DO runs the *Encrypt* function, taking the system public key *MPK*, the symmetric key *key*, and an LSSS access structure (M, ρ) as input, where *M* is an l × *n* matrix, and the function ρ maps attributes to the rows of the matrix *M*. The process is as follows: First, a random shared secret value $s\in Z_{p}^{*}$ is chosen, and a random vector $v=\left(s,\;y_{2},\;\ldots ,\;y_{n}\right)\in Z_{p}^{*}$ is generated. $\lambda_{i}=v\cdot M_{i}$ is computed, where i∈{1, …, l}, and *M_i_* is the *i*-th row vector of the matrix *M*. Then, random elements are chosen, and the computation process is shown in Equation (7).

(7) $$\begin{array}{c} C=\varepsilon \cdot e{\left(g,g\right)}^{a_{1}s},\tilde{C}=g^{s},\overset{⌢}{C}=g_{1}^{s},\\ \left(A_{1}=g^{a_{0}\lambda_{1}}{\left(g^{a_{2}}\right)}^{-r_{1}},B_{1}=g^{r_{1}}),\ldots ,(A_{l}=g^{a_{0}\lambda_{l}}{\left(g^{a_{2}}\right)}^{-r_{l}},B_{l}=g^{r_{l}}\right) \end{array}$$

The key ciphertext *CT* is obtained, as shown in Equation (8).

(8) $$CT=\{(M,\rho ),C,\tilde{C},\overset{⌢}{C},(A_{1},B_{1}),\ldots ,(A_{l},B_{l})\}$$

The obtained key ciphertext *CT* and data ciphertext *CF* are uploaded to the cloud storage system. Then, a smart contract is called to store the metadata $metadata=\{UFID,\;CT_{cid},\;CF_{cid},\;profile\}$ of the shared data in the blockchain system. Here, *CT_cid_* and *CF_cid_* are the storage addresses of the key ciphertext *CT* and the data ciphertext *CF* in the cloud storage system, respectively, and the profile is a brief introduction to the data file.

**Figure 4:**
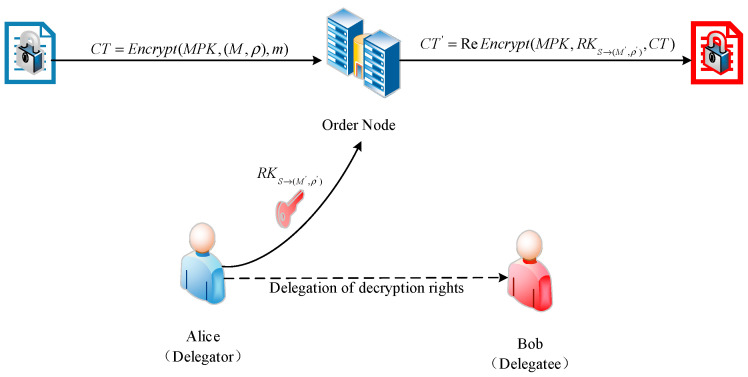
Re-encryption design.

3. Data Ciphertext Retrieval and Decryption. In the blockchain network, authorized users can freely query the metadata *metadata* and use the queried metadata to retrieve the corresponding key ciphertext *CT* and data ciphertext *CF* from the cloud storage system. For example, let Alice’s key be *USK_Alice_*. An authorized user calls the Decrypt function, which inputs the original key ciphertext. The specific process is as follows: For $I=\{i:\rho (i)\in S_{Alice}\}$ and *I* ⊆ {1, …, *l*}, if {*λ_i_*} is a valid share of the secret s according to the matrix *M*, and the user attribute set *S_1_* = {“*Attr1*”, “*Attr2*”, “*Attr6*: *3*”} is a subset of the weighted access policy *WT*, where the attributes “*Attr1*” and “*Attr2*” satisfy the (“*Attr1*” *AND* “*Attr2*”) policy, and the weight of “*Attr6*: *3*” is 3, which is greater than the minimum weight of “*Attr6*” in the access policy, i.e., 1. If the attribute set *S_Alice_* satisfies the access structure (M,ρ), i.e., *S_Alice_* | = (M,ρ), then there exists a constant set $\left\{\omega_{i}\in Z_{p}^{*}\right\}$ such that Equation (9) holds. The intermediate variable is computed using Equation (10).
  (9) $$\sum_{i\in I}\omega_{i}\cdot \lambda_{i}=s$$
  (10) $$\begin{array}{ll} \theta & =\frac{e(\tilde{C},K)}{\prod_{i\in I}{\left(e(A_{i},L)e(B_{i},T_{x})\right)}^{\omega_{i}}}\\ & =\frac{e(g^{s},g^{a_{0}\cdot h}g^{a_{1}})}{\prod_{i\in I}{\left(e(g^{a_{0}\lambda_{i}}{\left(g^{a_{2}}\right)}^{-r_{i}},g^{h})e(g^{r_{i}},g^{a_{2}h})\right)}^{\omega_{i}}}\\ & =\frac{e{\left(g,g\right)}^{a_{1}\cdot s}e{\left(g,g\right)}^{a_{0}\cdot h\cdot s}}{e{\left(g,g\right)}^{a_{0}\cdot h\cdot (\sum_{i\in I}\omega_{i}\cdot \lambda_{i})}}\\ & =e{\left(g,g\right)}^{a_{1}\cdot s} \end{array}$$
  Then, the symmetric key *key* is obtained using Equation (11).
  (11) $$\begin{array}{cc} H_{2}(C/\theta ) & =H_{2}(\varepsilon \cdot e{\left(g,g\right)}^{a_{1}s}/(e{\left(g,g\right)}^{a_{1}s}))\\ & =H_{2}(\varepsilon )\\ & =key \end{array}$$
  Finally, the data file *File* is output by running the symmetric decryption function, taking the key and *CF* as input.
4. Re-encryption of Ciphertext. When unauthorized users fail to decrypt, they cannot obtain the data file. In a blockchain network, when an unauthorized user attempts to obtain data, they first need to call a smart contract to acquire the metadata and then send a data-sharing request to an authorized user. This request information includes the metadata to be obtained and the unauthorized user’s *GID*. Upon receiving the request, if the authorized user agrees to share the data, they query the attribute information of the unauthorized user from the CA using their *GID*. A new weighted access policy *NWT*{“*GID_2_*” *AND* “*Attr6*:*1*” *AND* (“*Attr1*” *AND* “*Attr2*”)} is defined, where *GID_2_* is the globally unique identifier of the unauthorized user, and the access policy restricts access to only this user. As shown in Figure 4, the re-encryption key generation algorithm *reKeyGen* is run, taking the authorized user’s key *USK* and the new weighted access policy *NWT* as input and outputting the re-encryption key *RK*.

First, a random shared secret value $s^{\prime }\in Z_{p}^{*}$ is chosen, a random vector $v^{\prime }=(s^{\prime },y_{2}^{\prime },\ldots y_{n}^{\prime })\in Z_{p}^{*}$ is generated, and $\lambda_{i}^{\prime }=v^{\prime }\cdot M_{i}^{\prime }$ is computed, where $i\in \{1,\ldots ,l^{\prime }\}$ and $M_{i}^{\prime }$ are the *i*-th row vectors of the matrix $M^{\prime }$. Then, a random element $r_{1}^{\prime },\ldots ,r_{l^{\prime }}^{\prime }\in Z_{p}^{*}$ is chosen, $\partial \in G_{T}$ is randomly selected, and the computation steps are shown in Equation (12).

(12) $$\begin{array}{c} C^{\prime }=\partial \cdot e{\left(g,g\right)}^{\alpha s^{\prime }},{\tilde{C}}^{\prime }=g^{s^{\prime }},\\ \left(A_{1}^{\prime }=g^{a\lambda_{1}^{\prime }}{\left(g^{\beta }\right)}^{-r_{1}^{\prime }},B_{1}^{\prime }=g^{r_{1}^{\prime }}),\ldots ,(A_{l^{\prime }}^{\prime }=g^{a\lambda_{l}^{\prime }}{\left(g^{\beta }\right)}^{-r_{l}^{\prime }},B_{l^{\prime }}^{\prime }=g^{r_{l}^{\prime }}\right) \end{array}$$

A random element $\delta \in Z_{p}^{*}$ is selected, and the re-encryption key $RK_{s\to (M^{\prime },\rho^{\prime })}$ is computed using Equation (13).

(13) $$RK_{s\to (M^{\prime },\rho^{\prime })}=\left\{ \begin{array}{l} rk_{A}=K^{H_{2}(\partial )}g_{1}^{\delta },rk_{B}=g^{\delta },rk_{C}=L^{H_{2}(\partial )},\\ rk_{D}=((M^{\prime },\rho^{\prime }),C^{\prime },{\tilde{C}}^{\prime },A_{1}^{\prime },B_{1}^{\prime },\ldots ,A_{l^{\prime }}^{\prime },B_{l^{\prime }}^{\prime }),\\ rk_{E}=S_{Alice},\\ rk_{x}=T_{x}^{H_{2}(\partial )} \end{array}\right.$$

where $rk_{A}$ is a component K of the authorizer’s attribute key, calculated from the hash value $H_{2}(\partial )$, random element δ, and generator $g_{1}$, and $rk_{B}$ is derived from the generator *g* and random element δ. $rk_{C}$ is computed using the authorizer’s attribute key component *L* and hash value $H_{2}(\partial )$. $rk_{D}$ represents the new weighted access structure and the new ciphertext obtained from Equation (12). $rk_{E}$ denotes the authorizer’s attribute set. $rk_{x}$ is the result of calculations involving the authorizer’s attribute key component $T_{x}$ and hash value $H_{2}(\partial )$.

The authorized user then constructs a re-encryption request containing the re-encryption key *RK* and the metadata and sends it to the ordering node cluster. Upon receiving the re-encryption request from the authorized user, the ordering node cluster runs the re-encryption algorithm *reEncrypt*, taking the key ciphertext *CT* and the re-encryption key *RK* as input and outputting the re-encrypted ciphertext *RCT*.

The specific steps are as follows:

For $I=\{i:\rho (i)\in S_{Alice}\}$ and I⊆{1,…,l}, if $\left\{\lambda_{i}\right\}$ are valid shares of the secret *s* based on the matrix *M*, and the attribute set satisfies the access structure (M,ρ), i.e., $S_{Alice}|=(M,\rho )$, then there exists a set of constants $\left\{\omega_{i}\in Z_{p}^{*}\right\}$ such that Equation (9) holds. The ciphertext transformation component ϕ is obtained from Equation (14).

(14) $$\begin{array}{cc} \phi & =\frac{e(\tilde{C},rk_{A})/e(\overset{⌢}{C},rk_{B})}{\prod_{i\in I}{\left(e(A_{i},rk_{C})e(B_{i},rk_{\rho (i)})\right)}^{\omega_{i}}}\\ & =\frac{e(g^{s},{\left(g^{a\cdot h}g^{\alpha }\right)}^{H_{2}(\partial )}\cdot g_{1}^{\delta })/e(g_{1}^{s},g^{\delta })}{\prod_{i\in I}{\left(e(g^{a\lambda_{i}}{\left(g^{\beta }\right)}^{-r_{i}},g^{h\cdot H_{2}(\partial )})e(g^{r_{i}},{\left(g^{\beta }\right)}^{h\cdot H_{2}(\partial )})\right)}^{\omega_{i}}}\\ & =\frac{e(g^{s},g^{a\cdot h\cdot H_{2}(\partial )})e(g^{s},g^{\alpha \cdot H_{2}(\partial )})}{e{\left(g,g^{a\cdot h\cdot H_{2}(\partial )}\right)}^{\sum_{i\in I}\omega_{i}\lambda_{i}}}\\ & =e{\left(g,g\right)}^{\alpha \cdot H_{2}(\partial )\cdot s} \end{array}$$

The computation of the re-encrypted ciphertext *RCT* is obtained from Equation (15).

(15) $$RCT=\{C,\phi ,rk_{D}\}$$

The ordering node cluster returns the re-encrypted ciphertext *RCT* to the authorized user. Upon receiving *RCT*, the authorized user uploads it to the cloud storage system and calls the smart contract to store the address of *RCT*, as well as the re-encryption information, including the authorized user’s global identifier *GID*, the unauthorized user’s global identifier *GID*, the original ciphertext information, and the current timestamp, in the blockchain system. After obtaining the re-encrypted ciphertext *RCT*, the unauthorized user runs the decryption algorithm *reDecrypt* using their attribute private key, taking *RCT* and user Bob’s attribute key $SK_{Bob}$ as input. The specific steps of the algorithm are as follows:

For $I^{\prime }=\{i:\rho^{\prime }(i)\in S_{Bob}\}$ and $I^{\prime }\subseteq \{1,\ldots ,l^{\prime }\}$, if $\left\{\lambda_{i}^{\prime }\right\}$ are valid shares of the secret $s^{\prime }$ based on the matrix $M^{\prime }$, and the attribute set $S_{Bob}$ satisfies the access structure $\left(M^{\prime },\rho^{\prime }\right)$, i.e., $S_{Bob}|=(M^{\prime },\rho^{\prime })$, then there exists a set of constants $\left\{\omega_{i}^{\prime }\in Z_{p}^{*}\right\}$ such that $\sum_{i\in I^{\prime }}\omega_{i}^{\prime }\cdot \lambda_{i}^{\prime }=s^{\prime }$ holds. The value of the preceding variable $\theta^{\prime }$ is obtained from Equation (16).

(16) $$\begin{array}{cc} \theta^{\prime } & =\frac{e({\tilde{C}}^{\prime },K^{\prime })}{\prod_{i\in I^{\prime }}{\left(e(A_{i}^{\prime },L^{\prime })e(B_{i}^{\prime },T_{x}^{\prime })\right)}^{\omega_{i}^{\prime }}}\\ & =\frac{e(g^{s^{\prime }},g^{a\cdot h^{\prime }}g^{\alpha })}{\prod_{i\in I^{\prime }}{\left(e(g^{a\lambda_{i}^{\prime }}{\left(g^{\beta }\right)}^{-r_{i}^{\prime }},g^{h^{\prime }})e(g^{r_{i}^{\prime }},{\left(g^{\beta }\right)}^{h^{\prime }})\right)}^{\omega_{i}^{\prime }}}\\ & =\frac{e{\left(g,g\right)}^{\alpha \cdot s^{\prime }}e{\left(g,g\right)}^{a\cdot h^{\prime }\cdot s^{\prime }}}{e{\left(g,g\right)}^{a\cdot h^{\prime }\cdot (\sum_{i\in I^{\prime }}\omega_{i}^{\prime }\cdot \lambda_{i}^{\prime })}}\\ & =e{\left(g,g\right)}^{\alpha \cdot s^{\prime }} \end{array}$$

The essential secret value ∂ is obtained through Equation (17).

(17) $$\partial =\frac{C^{\prime }}{\theta^{\prime }}=\frac{\partial \cdot e{\left(g,g\right)}^{\alpha s^{\prime }}}{e{\left(g,g\right)}^{\alpha s^{\prime }}}$$

The symmetric key *key* is then computed using Equation (18).

(18) $$\begin{array}{cc} H_{2}(C/\phi^{\frac{1}{H_{2}(\partial )}}) & =H_{2}(\varepsilon \cdot e{\left(g,g\right)}^{\alpha s}/{\left(e{\left(g,g\right)}^{\alpha \cdot H_{2}(\partial )\cdot s}\right)}^{\frac{1}{H_{2}(\partial )}})\\ & =H_{2}(\varepsilon )\\ & =key \end{array}$$

Finally, the symmetric decryption function $D_{key}$ is run, taking the symmetric key *key* and the data ciphertext *CF* as inputs and outputting the data file *File*.

## 4. Results

The primary hardware environment consists of an Intel(R) Core(TM) i5-8250U CPU @ 1.60 GHz with 12 GB of RAM. The software environment utilizes Java for programming implementation, employing the Java Pairing-Based Cryptography (JPBC) library version 2.0.0. The experiments use a 160-bit elliptic curve group constructed from a 512-bit Type A supersingular curve defined by the equation y^2^ = x^3^ + x. Performance tests on WAMA-PRE are carried out while controlling the number of attribute authorities and attributes.

### 4.1. Performance Analysis with Different Number of Authorities

#### 4.1.1. Time Overhead

**Figure 5:**
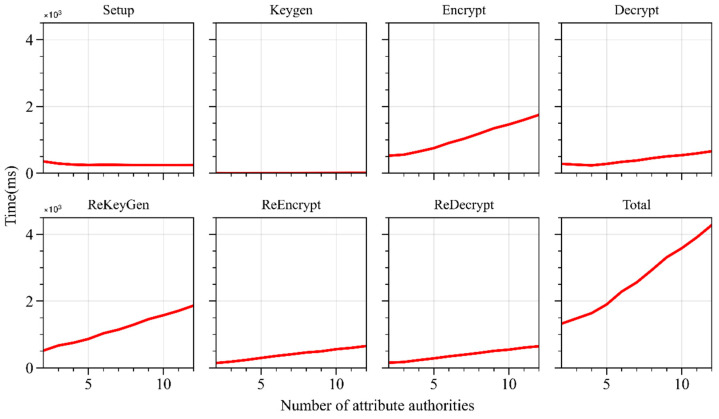
Algorithm running time with different numbers of attribute authorities.

With the number of attributes fixed at two, the number of authorities gradually increased from 2 to 12, with a step size of one. The experiment recorded the execution time, key size, and ciphertext size of WAMA-PRE under different numbers of authorities. As shown in Figure 5, as the number of authorities increases, the execution time of the proposed model’s *Setup* operation does not vary significantly, remaining around 240 ms. This is because the multiple attribute authorities execute the *Setup* in parallel. The *Keygen* operation time is short, less than 10 ms, and slightly increases because, as the number of communicating attribute authorities increases, the execution time of *Keygen* also gradually increases. The execution times of the *Encrypt* and *Decrypt* operations vary greatly, increasing linearly with the number of attribute authorities. The increased number of authorities leads to more complex access policies and, consequently, increased computation time. The time for *ReKeyGen*, *ReEncrypt*, and *ReDecrypt* operations also positively correlates with the number of authorities. As the number of authorities increases, computational complexity rises, leading to a notable increase in time overhead. Compared to user re-encryption operations, the proxy re-encryption operation saves 63% of time consumption.

#### 4.1.2. Space Overhead

**Figure 6:**
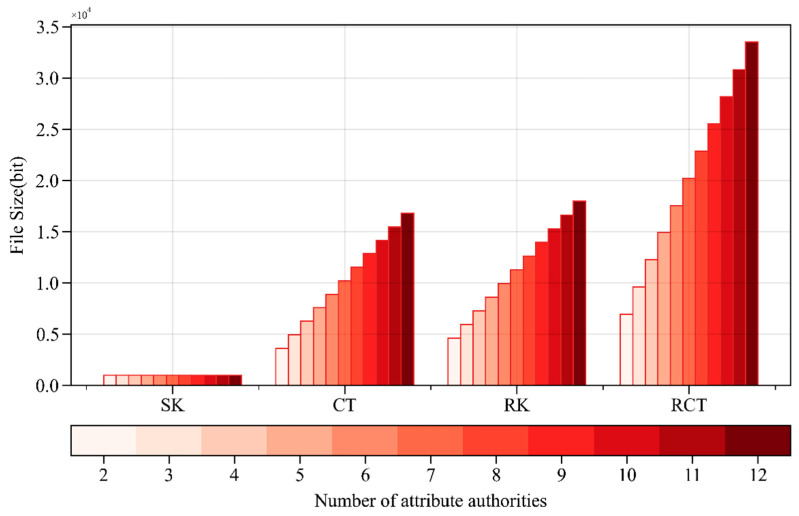
Storage space occupancy with different numbers of attribute authorities.

Figure 6 shows that, as the number of attribute authorities increases, the storage space occupied by the user’s private key remains around 994 bits, with slight variation. The user’s private key is obtained through group element multiplication. As the number of attribute authorities increases, the number of attributes in the access policy also gradually increases, and the storage space occupied by the ciphertext, which is closely related to the access policy, increases from 3634 bits to 16,816 bits. The sizes of the re-encryption key and re-encrypted ciphertext also slightly increase with the number of attribute authorities. The storage space occupied by the ciphertext, re-encryption key, and re-encrypted ciphertext are all positively correlated with the number of attribute authorities. However, the overall storage space occupied is relatively tiny.

#### 4.1.3. Scalability Analysis

As the number of attribute authorities increases, the *Setup* operation time remains constant, which is beneficial for system scalability, especially when there are numerous attribute authorities. The *Keygen* operation time increases slightly but does not exceed ten milliseconds at its peak. The time overhead for the *Encrypt*, *Decrypt*, *ReKeyGen*, *ReEncrypt*, and *ReDecrypt* operations positively correlates with the number of attribute authorities. Although the time overhead for these operations increases, the actual time consumption remains relatively low. The storage space occupied by ciphertexts, re-encryption keys, and re-encrypted ciphertexts also positively correlates with the number of attribute authorities, but the overall space occupation is small. These results indicate that the WAMA-PRE scheme performs well in terms of scalability.

### 4.2. Performance Analysis with Different Number of Attributes

#### 4.2.1. Time Overhead

**Figure 7:**
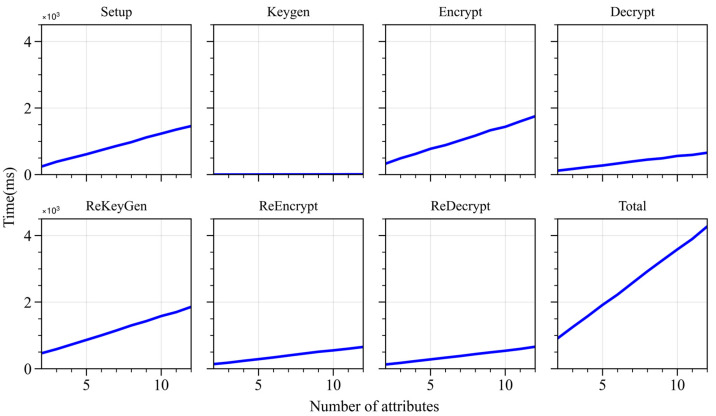
Algorithm running time with different numbers of attributes.

With the number of authorities fixed at two, the number of attributes was gradually increased from 2 to 12. The experiment recorded the execution time and key size for each operation as the number of attributes varied. As shown in Figure 7, as the number of attributes increases, the execution time of all operations increases accordingly. Specifically, the execution time of the *Setup* operation increases from 243 ms to 2504 ms; the execution time of the *Keygen* operation is minimal, with a maximum of only 14 ms; the execution time of the re-encryption algorithm is reduced by 63% compared to the encryption algorithm; and the execution time of the decryption algorithm is not significantly different from that of the re-decryption algorithm.

#### 4.2.2. Space Overhead

**Figure 8:**
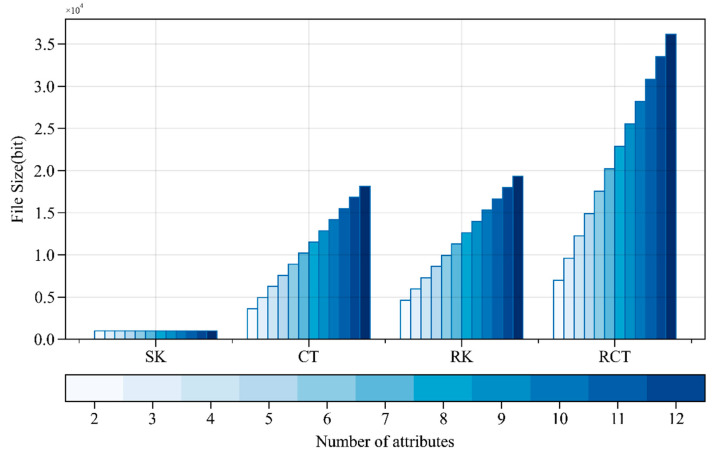
Storage space occupancy with different numbers of attributes.

Figure 8 shows that, as the number of attributes increases, the storage space occupied by the user’s key remains relatively stable. In contrast, the storage space occupied by the ciphertext, re-encryption key, and re-encrypted ciphertext gradually increases, with the ciphertext storage space increasing from 3639 bits to 27,383 bits. The sizes of the re-encryption key and ciphertext slightly increase with the number of attributes. The storage space occupied by the ciphertext, re-encryption key, and re-encrypted ciphertext are all positively correlated with the number of attribute authorities. However, the overall storage space occupied is relatively tiny.

#### 4.2.3. Scalability Analysis

As the number of attributes increases, the time overhead for the *Setup* operation continues to rise. However, considering that the Setup operation typically needs to be executed only once, this growth is within an acceptable range. It has a limited impact on the entire system’s real-time performance. The time overhead for the *Keygen* operation is minimal, with a maximum of only 14 ms, ensuring the scheme’s scalability regarding key generation. The growth in time overhead for the *Encrypt*, *Decrypt*, *ReKeyGen*, *ReEncrypt*, and *ReDecrypt* operations is also controllable. The storage space occupied by user private keys remains relatively stable, implying that the size of private keys is essentially unaffected by the increase in the number of attributes. The storage space occupied by ciphertexts increases with the number of attributes; nevertheless, considering modern storage technology advancements and network bandwidth advancements, this growth remains acceptable. The storage space occupied by re-encryption keys and re-encrypted ciphertexts also increases slightly with the number of attributes. However, the overall space occupation is small, indicating that the WAMA-PRE scheme has good scalability regarding storage requirements. The growth in time and space overhead of the WAMA-PRE scheme is within an acceptable range, making the scheme suitable for handling an increasing number of attributes and meeting the scalability requirements in practical applications.

## 5. Discussion

### 5.1. Quantitative Analysis

**Figure 9:**
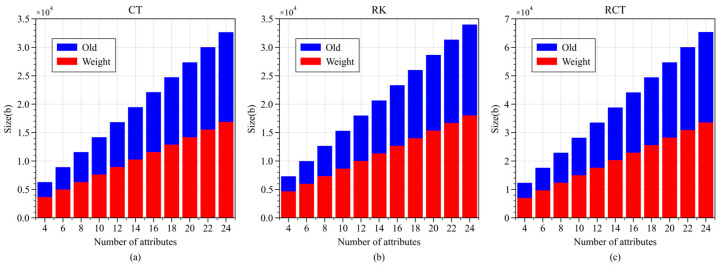
Storage space occupancy of weighted access policy vs. ordinary access policy. (**a**) Ciphertext; (**b**) re-encryption key; (**c**) re-encrypted ciphertext.

WAMA-PRE employs a weighted access policy, which, compared to traditional unweighted access policies, offers the advantages of more concise expression and lower storage space utilization. To discuss the difference between the two in terms of storage space usage, as illustrated in Figure 9, the weighted access policy (Weight) demonstrates a 50% reduction in storage space occupation compared to the traditional unweighted access policy (Old).

To further analyze and compare the performance of WAMA-PRE, this study conducted comparative experiments with the schemes proposed by Yang [43] and Banerjee [44]. Under controlled numbers of attribute authorities and attributes, the performance of different schemes was tested, with all thresholds in the access structure set to AND, representing the worst-case scenario for the algorithm.

**Figure 10:**
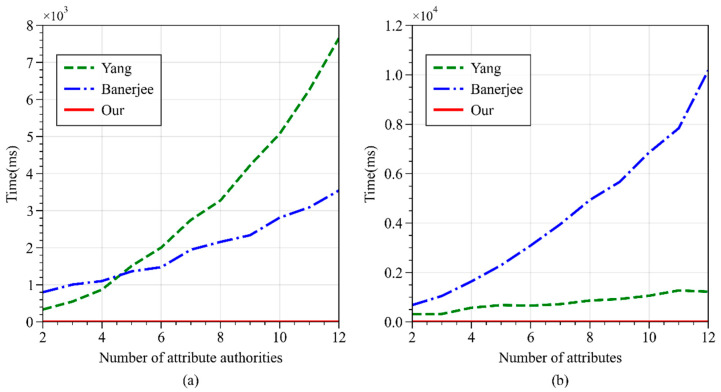
Time consumption of Keygen operations in different schemes. (**a**) With fixed number of attributes; (**b**) with fixed number of authorities.

As shown in Figure 10, when the number of attributes is fixed, and the number of authorities is gradually increased, the time efficiency of Banerjee’s scheme is better than Yang’s scheme. However, when the number of authorities is fixed, and the number of attributes is gradually increased, the time efficiency of Yang’s scheme is better than Banerjee’s scheme. Regardless of whether the number of attribute authorities or attributes is controlled, the execution time of WAMA-PRE’s *KeyGen* operation is significantly lower than the other two schemes.

**Figure 11:**
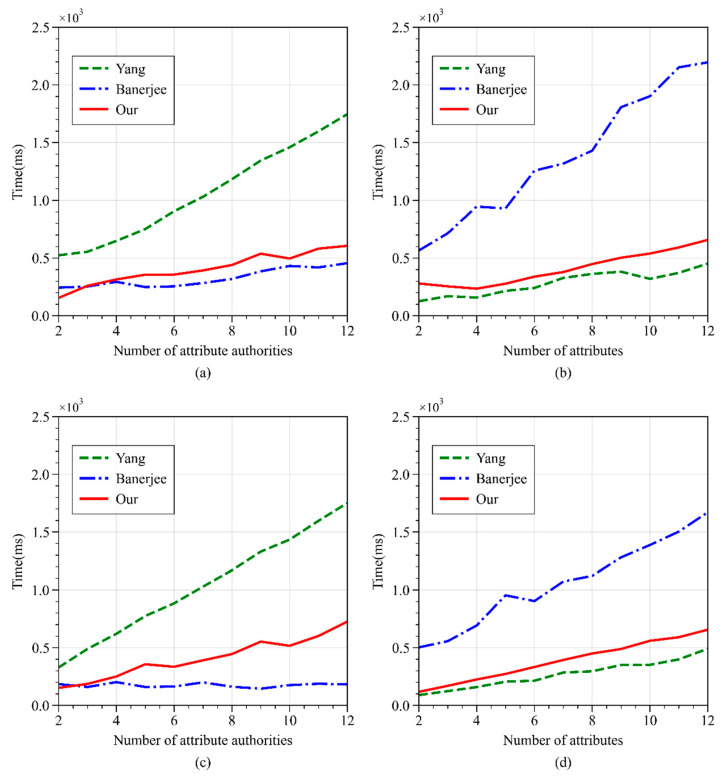
Time consumption of encryption and decryption operations in different schemes. (**a**) Encryption operation with fixed number of attributes; (**b**) encryption operation with fixed number of authorities; (**c**) decryption operation with fixed number of attributes; (**d**) decryption operation with fixed number of authorities.

This study compared the execution times of the Encrypt and Decrypt operations for the three schemes under controlled numbers of attribute authorities and attributes. As shown in Figure 11a, as the number of authorities increases, the encryption time of all three schemes grows linearly, with WAMA-PRE’s time consumption lower than Yang’s scheme but slightly higher than Banerjee’s scheme. Figure 11b shows that, as the number of authorities increases, the decryption time of WAMA-PRE is close to Yang’s scheme and lower than Banerjee’s scheme. Figure 11c shows that, as the number of attributes increases, the encryption time of WAMA-PRE is lower than Yang’s scheme but slightly higher than Banerjee’s scheme. As shown in Figure 11d, as the number of attributes increases, the decryption time of WAMA-PRE is slightly higher than Yang’s scheme.

Overall, the time cost of encryption and decryption in WAMA-PRE is lower than Banerjee’s scheme but slightly higher than Yang’s scheme. This is because WAMA-PRE supports proxy re-encryption, which requires additional computations. However, proxy re-encryption saves significant time and space in subsequent access policy updates.

**Figure 12:**
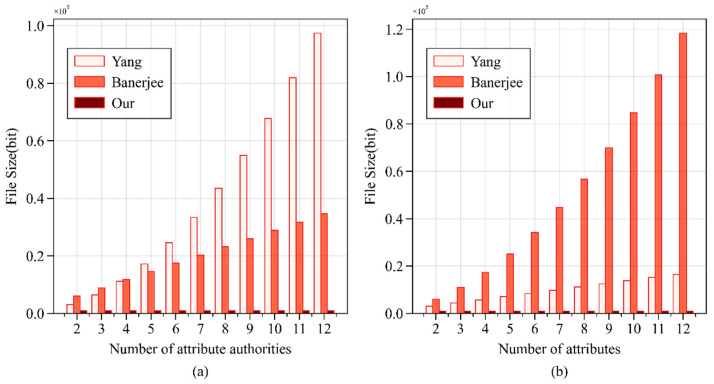
Storage space occupancy in different schemes. (**a**) With fixed number of attributes; (**b**) with fixed number of authorities.

This study analyzed the storage space occupied by the private keys of different schemes, with the results shown in Figure 12. Regardless of whether the number of attribute authorities or attributes is controlled, the storage space occupied by WAMA-PRE’s private key is the smallest. Specifically, when the number of authorities is fixed, Yang’s scheme occupies the most significant storage space; when the number of attributes is fixed, Banerjee’s scheme occupies the most significant storage space.

**Figure 13:**
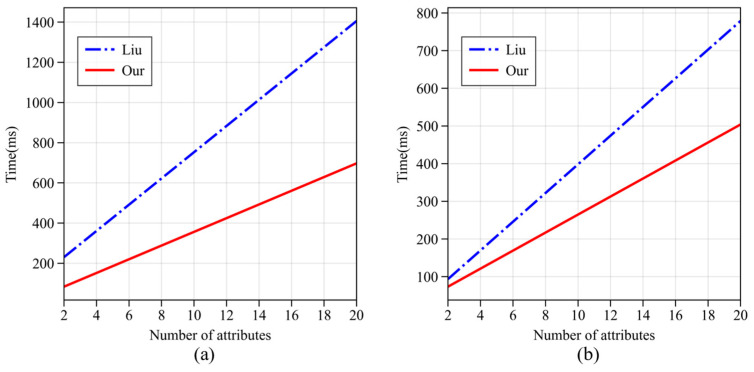
Computational efficiency comparison of re-encryption and encryption operations. (**a**) Re-encryption algorithm; (**b**) re-decryption algorithm.

A comparison was made with the scheme proposed by Liu et al. [35], with the results shown in Figure 13. Figure 13a shows that the computational efficiency of the re-encryption algorithm in this paper’s scheme is 53% higher than that of Liu’s scheme, and Figure 13b shows that the computational efficiency of the re-decryption algorithm in this paper’s scheme is 32% higher than that of Liu’s scheme.

### 5.2. Theoretical Analysis

#### 5.2.1. Functional Comparison

This study compared and analyzed the functionality of WAMA-PRE with Yang’s and Banerjee’s schemes, with the results shown in Table 2. It can be seen that, although Yang’s and Banerjee’s schemes also introduced multiple attribute authority centers, they cannot realize the re-encryption function. Additionally, the WAMA-PRE scheme adopts the LSSS access structure, enabling more flexible access policies without affecting efficiency, and introduces a weighted access policy, which, with the same access control effect, results in a more concise access policy and lower ciphertext space usage.

#### 5.2.2. Storage Space Comparison

This study compared the storage space of WAMA-PRE with Yang’s and Banerjee’s schemes. For convenience of description, |*G*|, |*G_T_*|, and |$Z_{p}$| represent the lengths of elements in groups *G*, *G_T_*, and $Z_{p}^{*}$, respectively, and n is the number of attributes. As listed in Table 3, compared to other schemes, the system public key, system master key, and user key in this paper’s system have significant advantages in terms of storage overhead.

#### 5.2.3. Security Model Discussion

This study uses the AES symmetric encryption algorithm to encrypt plaintext information and employs a multi-authority weighted attribute-based proxy re-encryption algorithm to encrypt the symmetric key. Therefore, it is only necessary to provide security proof for the multi-authority weighted attribute-based proxy re-encryption. This paper defines a selective access structure and chosen-plaintext attack (SAS-CPA) security game between an adversary 𝓡 and a challenger 𝓒, with the following specific steps:

**Initialization:** the adversary 𝓡 selects a weighted access structure (M,ρ) and sends it to the challenger 𝓒.

**Setup:** The challenger 𝓒 runs *GlobalSetup* to output the system master key *MSK* and public key *MPK*. The system public key *MPK* is then sent to the adversary 𝓡. For each attribute *attr_j_*, the attribute authority *AA_i_* randomly selects $h_{i,j}\in Z_{p}^{*}$ as the key *SK_i,j_* and generates the attribute public key *PK_i,j_*.

**Query Phase I:** The adversary 𝓡 makes the following queries, and 𝓒 responds according to the following rules:

(1) Attribute key extraction query $Q_{SK}(S_{1}^{*})$: given an attribute set *S*_1_^*^, 𝓒 obtains the user key components from each attribute authority, runs the *KeyGen* algorithm to generate the user key *USK*_𝓡_, and sends it to 𝓡.

(2) Re-encryption key extraction query $Q_{RK}(S_{1}^{*},(M,\rho ))$: given an attribute set *S*_2_^*^ and a new weighted access structure (M, ρ), where *S*_2_^*^| ≠ (*M*, *ρ*), 𝓒 runs the *ReKeyGen* algorithm to generate the re-encryption key $RK_{S_{2}^{*}\to (M,\rho )}$ and then sends $RK_{S_{2}^{*}\to (M,\rho )}$ to 𝓡, where $S_{1}^{*}|\neq (M^{*},\rho^{*})$, $S_{2}^{*}|\neq (M^{*},\rho^{*})$.

**Challenge:** 𝓡 selects two equal-length messages $m_{0}$ and $m_{1}$ and sends them to 𝓒. 𝓒 randomly selects σ∈{0,1}, runs the Encrypt algorithm with input $m_{\sigma }$, the weighted access structure (M, ρ), and the system public key *MPK* to generate the ciphertext *CT*, and then it returns *CT* to 𝓡.

**Query Phase II:** 𝓡 repeats the operations of **Query Phase I**.

**Guess:** 𝓡 guesses $\sigma^{*}\in \{0,1\}$. If $\sigma^{*}=\sigma$, then 𝓡 wins the game. As shown in Equation (19), the advantage of 𝓡 winning the game is calculated.

(19) $$Adv_{ℜ}^{sAS-CCB-CP-ABPRE}(1^{k})=|\mathrm{Pr}[\sigma^{*}=\sigma ]-\frac{1}{2}|$$

#### 5.2.4. Security Proof

**Definition** **1.** *If an adversary can only win the SAS-CPA security game with a negligible advantage in any probabilistic polynomial time, the scheme is indistinguishable under a selective access structure and chosen-plaintext attack; i.e., the scheme is provably indistinguishable under the selective access structure and chosen-plaintext attack (IND-SAS-CPA) secure in the random oracle model.*

**Theorem** **1.** *If an adversary 𝓡 can win the SAS-CPA security game with a non-negligible advantage μ in any probabilistic polynomial time, then there exists a challenger 𝓒 that can solve the decisional q-parallel BDHE problem with an advantage of μ/2.*

**Proof of Theorem** **1.** In the SAS-CPA security game, the challenger 𝓒 chooses two multiplicative cyclic groups G and $G_{T}$ of prime order p, a random generator g∈G, a bilinear map $e:G\times G\to G_{T}$, a q-parallel BDHE instance ξ, and T.

**Initialization:** 𝓡 sends the challenged weighted access structure (M^*^, ρ^*^) to 𝓒, where M^*^ is an l^*^ × n^*^ matrix, $l^{*},n^{*}\leq q$.

**Setup:** 𝓒 chooses $\alpha_{1}^{\prime },\;\gamma \in Z_{p}^{*}$ and sets $g_{1}=g^{\gamma }$, $e{\left(g,g\right)}^{a_{1}}=e(g^{a_{1}},g^{a_{1}^{q}})\cdot e(g,g^{a_{1}\prime })$. 𝓒 selects hash functions *H*_1_ and *H*_2_ and sends the system public key $MPK=\{p,G,G_{T},g_{1},g,g^{a_{0}},e,H_{1},H_{2},e{\left(g,g\right)}^{a_{1}},g^{a_{2}}\}$ to 𝓡. 𝓒 simulates random oracles $H_{j}(j\in \{1,2\})$, controlled by 𝓒. If 𝓡 queries *H_j_*, 𝓒 responds according to the following rules:

***H*_1_:** For a query on $x\in U_{AA_{i}}$ ($U_{AA_{i}}$ is the set of all attributes of attribute authority $AA_{i}$), if there exists a tuple $\left(x,\;z_{x},\;\partial_{2,x}\right)$ in the *H*_1_ list, 𝓒 returns the existing $\partial_{2,x}$ to 𝓡, where $z_{x}\in Z_{p}^{*},\partial_{2,x}\in G$. Otherwise, 𝓒 constructs $\partial_{2,x}$ as follows: Let *X* be the set of indices *i* such that ρ^*^(*i*) = *x*. *X* contains the same attribute *x* corresponding to the row labels in matrix M^*^. 𝓒 chooses $z_{x}\in Z_{p}^{*}$ and sets the value of $\partial_{2,x}$ as shown in Equation (20).

(20) $$\partial_{2,x}=g^{z_{x}}\cdot \prod_{i\in X}g^{a_{1}\cdot M_{i,1}^{*}/b_{i}+a_{1}^{2}\cdot M_{i,2}^{*}/b_{i}+\ldots +a_{1}^{n^{*}}\cdot M_{i,n^{*}}^{*}/b_{i}}$$

If *X* is empty, 𝓒 sets $\partial_{2,x}=g^{z_{x}}$. 𝓒 returns $\partial_{2,x}$ to 𝓡 and adds the tuple $\left(x,\;z_{x},\;\partial_{2,x}\right)$ to the *H*_1_ list.

***H*_2_:** For a query on $\partial \in G_{T}$, if a tuple $\left(\partial ,b^{*}\right)$ exists in the *H*_2_ list, 𝓒 sends the existing value b^*^ to 𝓡, where $b\in Z_{p}^{*}$. Otherwise, 𝓒 sets $H_{2}(\partial )=b^{*}$, returns b^*^ to 𝓡, and adds the tuple $\left(\partial ,b^{*}\right)$ to the *H*_2_ list.

**Query Phase I:** 𝓡 makes a series of queries to 𝓒, and 𝓒 responds according to the following rules:

Secret Key Query $Q_{SK}(S_{1}^{*})$: 𝓡 constructs a user secret key *USK_𝓡_* for the attribute set *S*_1_^*^ as follows: If *S*_1_^*^| = (M^*^, ρ^*^), 𝓒 randomly outputs {0, 1} and aborts the game. Otherwise, 𝓒 chooses a random element $r_{s}\in Z_{p}^{*}$ and finds a vector $\omega =(\omega_{1},\ldots ,\omega_{n*})\in Z_{p}^{*}$ such that $\omega_{1}=-1$ and $\omega \cdot M^{*}=0$ for $\forall i,\rho^{*}(i)\in S_{1}^{*}$.

𝓒 sets *L*, as shown in Equation (21).

(21) $$L=g^{r_{S}}\cdot \prod_{i=1,\ldots ,n^{*}}g^{a_{1}^{q+1-i}\cdot \omega_{i}}=g^{h}$$

Then, 𝓒 constructs *K* using Equation (22) and obtains a valid verification for *K* using Equation (23).

(22) $$K=g^{a_{1}^{\prime }}\cdot g^{a_{0}r_{S}}\cdot \underset{i=2,\ldots ,n^{*}}{\prod }g^{a_{0}^{q+2-i}\cdot \omega_{i}}$$

(23) $$\begin{array}{cc} K & =g^{a_{1}^{\prime }}\cdot g^{a_{0}r_{S}}\cdot \underset{i=2,\ldots ,n^{*}}{\prod }g^{a_{0}^{q+2-i}\cdot \omega_{i}}\\ & =g^{a_{1}^{\prime }}\cdot g^{a_{0}^{q+1}}\cdot g^{-a_{0}^{q+1}}\cdot g^{a_{0}r_{S}}\cdot \underset{i=2,\ldots ,n^{*}}{\prod }g^{a_{0}^{q+2-i}\cdot \omega_{i}}\\ & =g^{a_{1}}\cdot {\left(g^{r_{S}}\cdot \underset{i=1,\ldots ,n^{*}}{\prod }g^{a_{0}^{q+1-i}}\right)}^{a_{0}}\\ & =g^{a_{1}}\cdot g^{a_{0}h} \end{array}$$

If *x*∈*S*_1_^*^, and for all $i\in \{1,\ldots ,l^{*}\}$, $\rho^{*}(i)\neq x$, 𝓒 sets $T_{x}=L^{z_{x}}$. Then, T is computed as shown in Equation (24) if *X* is non-empty or as shown in Equation (25).

(24) $$T_{x}={\left(g^{a_{2}h}\right)}^{z_{x}}=\partial_{2,x}^{h}$$

(25) $$\begin{array}{cc} T_{x} & =L^{z_{x}}\cdot {\prod_{i\in X}\prod_{j=1,\ldots ,n^{*}}\left(g^{(a_{1}^{j}/b_{i})\cdot r_{S}}\cdot \prod_{k=1,\ldots ,n^{*},k\neq j}{\left(g^{a_{1}^{q+1+j-k}/b_{i}}\right)}^{\omega_{k}}\right)}^{M_{i,j}^{*}}\\ & =L^{z_{x}}\cdot {\prod_{i\in X}\prod_{j=1,\ldots ,n^{*}}\left(g^{(a_{1}^{j}/b_{i})\cdot r_{S}}\cdot \prod_{k=1,\ldots ,n^{*},k\neq j}{\left(g^{a_{1}^{q+1+j-k}/b_{i}}\right)}^{\omega_{k}}\right)}^{M_{i,j}^{*}}\cdot {\prod_{i\in X}\prod_{j=1,\ldots ,n^{*}}\left(g^{q+1/b_{i}}\right)}^{\omega_{j}\cdot M_{i,j}^{*}}\\ & ={\left(g^{r_{S}}\cdot \prod_{i=1,\ldots ,n^{*}}g^{a_{1}^{q+1-i}\cdot \omega_{i}}\right)}^{Z_{x}}\cdot {\prod_{i\in X}\prod_{j=1,\ldots ,n^{*}}\left(g^{(a_{1}^{j}/b_{i})\cdot r_{S}}\cdot \prod_{k=1,\ldots ,n^{*}}{\left(g^{a_{1}^{q+1+j-k}/b_{i}}\right)}^{\omega_{k}}\right)}^{M_{i,j}^{*}}\\ & ={\left(g^{z_{x}}\cdot \prod_{i\in X}g^{a_{1}\cdot M_{i,1}^{*}/b_{i}+a_{1}^{2}\cdot M_{i,2}^{*}/b_{i}+\ldots +a_{1}^{n^{*}}\cdot M_{i,n^{*}}^{*}/b_{i}}\right)}^{r_{S}+\omega_{1}\cdot a_{1}^{q}+\ldots +\omega_{n^{*}}\cdot a_{1}^{q-n^{*}+1}}\\ & =\partial_{2,x}^{r_{S}+\omega_{1}\cdot a_{1}^{q}+\ldots +\omega_{n^{*}}\cdot a_{1}^{q-n^{*}+1}}=\partial_{2,x}^{a_{2}\prime }=g^{a_{2}h} \end{array}$$

If *S*_1_^*^|≠ (M^*^, ρ^*^), the vector ω is obtained such that *ω*·M^*^ = 0, allowing the expression in Equation (25) to be computed as shown in Equation (26).

(26) $${\prod_{i\in X}\prod_{j=1,\ldots ,n^{*}}\left(g^{q+1/b_{i}}\right)}^{\omega_{j}\cdot M_{i,j}^{*}}=g^{a_{1}^{q+1}\cdot (\sum_{i\in X}\sum_{j=1,\ldots ,n^{*}}\omega_{j}\cdot M_{i,j}^{*}/b_{i})}=g^{0}=1$$

Finally, 𝓒 adds the tuple ($S_{1}^{*}$, *USK_𝓡_*) to the list and sends *USK_𝓡_* to 𝓡.

Re-encryption Key Query $Q_{RK}(S_{2}^{*},(M,\rho ))$: 𝓡 queries $Q_{RK}$ with an attribute set *S*_2_^*^ and a weighted access structure (*M*, *ρ*). If *S*_2_^*^| ≠ (M^*^, ρ^*^), 𝓒 first runs $Q_{RK}$ to obtain a user secret key and then outputs a re-encryption key $RK_{S_{2}^{*}\to (M,\rho )}$ in two steps:
**Step 1:** 𝓒 chooses $\delta ,b\in Z_{p}^{*},\bar{K}\in G$. 𝓒 computes the re-encryption key $rk_{A}=\bar{K}\cdot g_{1}^{\delta },rk_{B}=g^{\delta },rk_{C}=g^{b},rk_{x}=\partial_{2,x}^{b},rk_{E}=S_{2}^{*}$, where $\partial_{2,x}^{b}$ is the output of querying *H*_1_ on *x*, for $x\in S_{2}$, and constructs $rk_{D}$.
**Step 2:** 𝓒 returns $RK_{S_{2}^{*}\to (M,\rho )}=(rk_{A},rk_{B},rk_{C},rk_{D},rk_{E},rk_{x})$ to 𝓡; otherwise, 𝓒 randomly outputs {0, 1} and aborts the game.
**Challenge:** 𝓡 sends two equal-length messages $m_{0}$, $m_{1}$ to 𝓒. 𝓒 randomly chooses σ∈{0, 1} and responds as follows: For each row *i* of M^*^, 𝓒 sets x^*^ = ρ^*^(*i*) and queries $H_{1}$ on x^*^ to obtain the tuple $\left(x,\;z_{x},\;\partial_{2,x}\right)$. 𝓒 chooses $y_{2}^{\prime },\ldots ,y_{n^{*}}^{\prime }\in Z_{p}^{*}$ and uses vector sharing to share the secret $v=(s,s\cdot a_{1}+y_{2}^{\prime },s\cdot a_{1}^{2}+y_{3}^{\prime },\ldots ,s\cdot a_{1}^{n^{*}-1}+y_{n^{*}}^{\prime })\in Z_{p}^{*}$. For all $i\in \{1,\ldots ,l^{*}\}$, where *R_i_* is the set of all *i* ≠ *k* but ρ^*^(*i*) = ρ^*^(*k*). 𝓒 sets A_i_^*^ and B_i_^*^ using Equations (27) and (28).
  (27) $$A_{i}^{*}=\partial_{2,x}^{-r_{i}^{\prime }}\cdot (\prod_{j=2,\ldots ,n^{*}}g^{a_{1}\cdot M_{i,j}^{*}\cdot y_{j}^{\prime }})\cdot g^{b_{i}\cdot s\cdot (-z_{x}^{*})}\cdot {\left(\prod_{k\in R_{i}}\prod_{j=1,\ldots ,n^{*}}{\left(g^{a_{1}^{j}\cdot s\cdot (b_{i}/b_{k})}\right)}^{M_{k,j}^{*}}\right)}^{-1}$$
  (28) $$B_{i}^{*}=g^{r_{i}^{\prime }+s\cdot b_{i}}$$
  It is defined that $T\cdot e(g^{s},g^{{a^{\prime }}_{1}})=C^{*}/m_{\sigma }$ and ${\tilde{C}}^{*}=g^{s},{\overset{⌢}{C}}^{*}=g_{1}^{s}$. 𝓒 outputs the challenge ciphertext $CT^{*}=((M^{*},\rho^{*}),C^{*},{\tilde{C}}^{*},{\overset{⌢}{C}}^{*},(A_{1}^{*},B_{1}^{*}),\ldots ,(A_{l^{*}}^{*},B_{l^{*}}^{*}))$ and sends it to 𝓡.
If $T=e{\left(g,g\right)}^{a_{1}^{q+1}\cdot s}$, then CT^*^ is a valid ciphertext.
**Query Phase II:** 𝓡 repeats the operations of **Query Phase I**.
**Guess:** 𝓡 guesses σ*∈{0, 1}. If $\sigma^{*}=\sigma$, then 𝓒 outputs one and obtains $T=e{\left(g,g\right)}^{a_{1}^{q+1}\cdot s}$; otherwise, 𝓒 outputs 0, where *T* is a random element $R\in G_{T}$. The following process calculates the probability of 𝓒’s success.
When the output is 1, i.e., $T=e{\left(g,g\right)}^{a_{1}^{q+1}\cdot s}$, 𝓡 obtains a valid ciphertext. It is known that 𝓡 can win the game with a non-negligible advantage, so $\mathrm{Pr}[\sigma^{*}=\sigma |T=e{\left(g,g\right)}^{a_{1}^{q+1}\cdot s}]=\frac{1}{2}+\mu$.
When the output is 0, i.e., *T* = *R*, 𝓡 cannot obtain a valid ciphertext, so $\mathrm{Pr}[\sigma^{*}=\sigma |T=R]=1/2$. Therefore, 𝓒’s advantage in solving the decisional q-parallel BDHE problem is calculated as shown in Equation (29).
  (29) $$\begin{array}{ll} Adv_{C}^{q-parallel\;BDHE} & =|\mathrm{Pr}[\sigma^{*}=\sigma ]-\frac{1}{2}|\\ & =|\frac{1}{2}\mathrm{Pr}[\sigma^{*}=\sigma |\sigma =0]+\frac{1}{2}\mathrm{Pr}[\sigma^{*}=\sigma |\sigma =1]-\frac{1}{2}|\\ & =\frac{\mu }{2} \end{array}$$
  In summary, if 𝓡 can break the scheme with a non-negligible advantage *μ*, then 𝓒 can solve the decisional q-parallel BDHE problem with an advantage of *μ*/2, which contradicts the known hardness assumption. Therefore, the WAMA-PRE scheme is IND-sAS-CPA secure in the random oracle model. □

However, it is worth noting that the WAMA-PRE scheme is designed and experimented with only in a single blockchain system, and its limitation lies in its inability to be directly applied to a multi-chain environment. A multi-chain environment involves interaction and collaboration between multiple blockchain networks, posing higher complexity and unique challenges, such as cross-chain communication, data consistency, and security [45]. Future research should focus on extending the scheme to support a multi-chain environment, ensuring its effectiveness and reliability in a more complex blockchain ecosystem.

## 6. Conclusions

This study proposes a multi-authority weighted attribute-based proxy re-encryption scheme by combining blockchain technology and attribute-based proxy re-encryption techniques and designs a blockchain-based access control method for cloud storage data. The scheme utilizes attribute-based encryption technology to solve cloud storage data’s privacy and security issues while enabling data sharing and fine-grained access control. Using proxy re-encryption techniques, the scheme addresses the computational problem of repeatedly encrypting public key information on the blockchain, reducing user overhead and enabling delegated authorization. Introducing attribute weights makes access policies more flexible and reduces the storage space occupied by ciphertexts. The blockchain network composed of multiple attribute authorities enhances the scheme’s availability. The joint key construction between attribute authorities and the authorization center weakens the authority of the authorization center. This study provides a promising solution for secure data sharing in cloud storage.

## Figures and Tables

**Table 1:** Cryptographic symbols used in the scheme.

Symbol	Description
*MSK*	System master key
*MPK*	System public key
*USK*	User key
*key*	Symmetric encryption key
*CF*	Symmetric encryption ciphertext
*CT*	Key ciphertext
LSSS	Linear secret sharing scheme
*RK*	Re-encryption key
*RCT*	Re-encrypted ciphertext

**Table 2:** Functionality comparison of schemes.

Scheme	Attribute Encryption	Proxy Re-Encryption	Weighted Access Policy	Multiple Attribute Authorities	Access Structure
Li [7]	YES	NO	Yes	NO	Access Tree
Yang [43]	YES	NO	NO	YES	Access Tree
Banerjee [44]	YES	NO	NO	YES	Access Tree
Liu [35]	YES	YES	NO	Yes	Access Tree
Our	YES	YES	YES	YES	LSSS

**Table 3:** Storage space comparison.

Scheme	System Public Key	System Master Key	User Secret Key	Ciphertext
Yang [43]	|*G*| + |*Z_p_*|	|*Z_p_*|	|*G*|·(1 + *n*)	|*G*|·(1 + *n*) + |*G_T_*|
Banerjee [44]	|*G_T_*| + 4|*G*|	|*Z_p_*|	|*G*|·(1 + *n*)	|*G*| + 2|*G_T_*| + |*Z_p_*|
Our	|*G_T_*| + |*G*|	2|*Z_p_*|	3|*G*|	2|*G*|·(1 + *n*) + |*G_T_*|

## Data Availability

The code and data for this study experiment can be downloaded and accessed through the following link: https://github.com/bob520/WAMA-PRE (accessed on 27 June 2024).
